# Predictive performance of the TRI-SCORE in patients with severe aortic stenosis and concomitant tricuspid regurgitation undergoing TAVR

**DOI:** 10.1007/s00392-025-02671-y

**Published:** 2025-05-12

**Authors:** Mustafa Mousa Basha, Baravan Al-Kassou, Christopher Gestrich, Marcel Weber, Thomas Beiert, Sebastian Zimmer, Farhad Bakhtiary, Georg Nickenig, Jasmin Shamekhi

**Affiliations:** 1 Heart Center, Department of Medicine II, University Hospital Bonn, Venusberg-Campus 1, 53127 Bonn, Germany; 2 Heart Center Bonn, Department of Cardiac Surgery, University Hospital Bonn, Bonn, Germany

**Keywords:** Aortic valve stenosis, Transcatheter aortic valve replacement, Tricuspid regurgitation, TRI-SCORE, EuroSCORE II, STS-Score

## Abstract

**Background:**

Tricuspid regurgitation (TR) is a common comorbidity in patients with severe aortic stenosis (AS) undergoing transcatheter aortic valve replacement (TAVR) and represents a significant predictor of adverse outcomes. Precise risk stratification through clinical scoring systems is vital for tailoring treatment decisions in this patient population.

**Objectives:**

To assess the applicability of the TRI-SCORE for predicting adverse outcomes in patients with AS and concomitant moderate-to-severe TR undergoing TAVR and to compare its performance with established surgical risk scores like the EuroSCORE II and Society of Thoracic Surgeons score (STS-Score).

**Methods:**

We conducted a retrospective analysis of 301 patients with severe AS and concomitant TR who underwent TAVR between 2013 and 2022 at the Heart Center Bonn. According to the TRI-SCORE, patients were stratified into a low or intermediate-risk group (TRI-SCORE 0–5) and a high-risk group (TRI-SCORE 6–12). The primary endpoint was 2-year all-cause mortality. Predictive values of the TRI-SCORE were compared to the EuroSCORE II and the STS-Score for both 30-day and 2-year mortality outcomes.

**Results:**

The 2-year mortality rate was significantly higher in the high-risk group compared to the low or intermediate-risk group (TRI-SCORE 6–12: 40.0% vs. TRI-SCORE 0–5: 17.9%; *p* < 0.001). For predicting 30-day mortality, the EuroSCORE II and the STS-Score demonstrated superior predictive values, with AUCs of 78.4% and 83.0%, respectively, in comparison to the TRI-SCORE, which showed an AUC of 70.0%. Conversely, the TRI-SCORE allowed a better risk prediction with regard to 2-year all-cause mortality, achieving an AUC of 69.7%, superior to the EuroSCORE II (60.6%) and the STS-Score (62.1%).

**Conclusion:**

The TRI-SCORE is effective in predicting mid-term mortality in patients with AS and moderate-to-severe TR undergoing TAVR, demonstrating greater robustness than the EuroSCORE II and the STS-Score for this timeframe.

**Graphical abstract:**

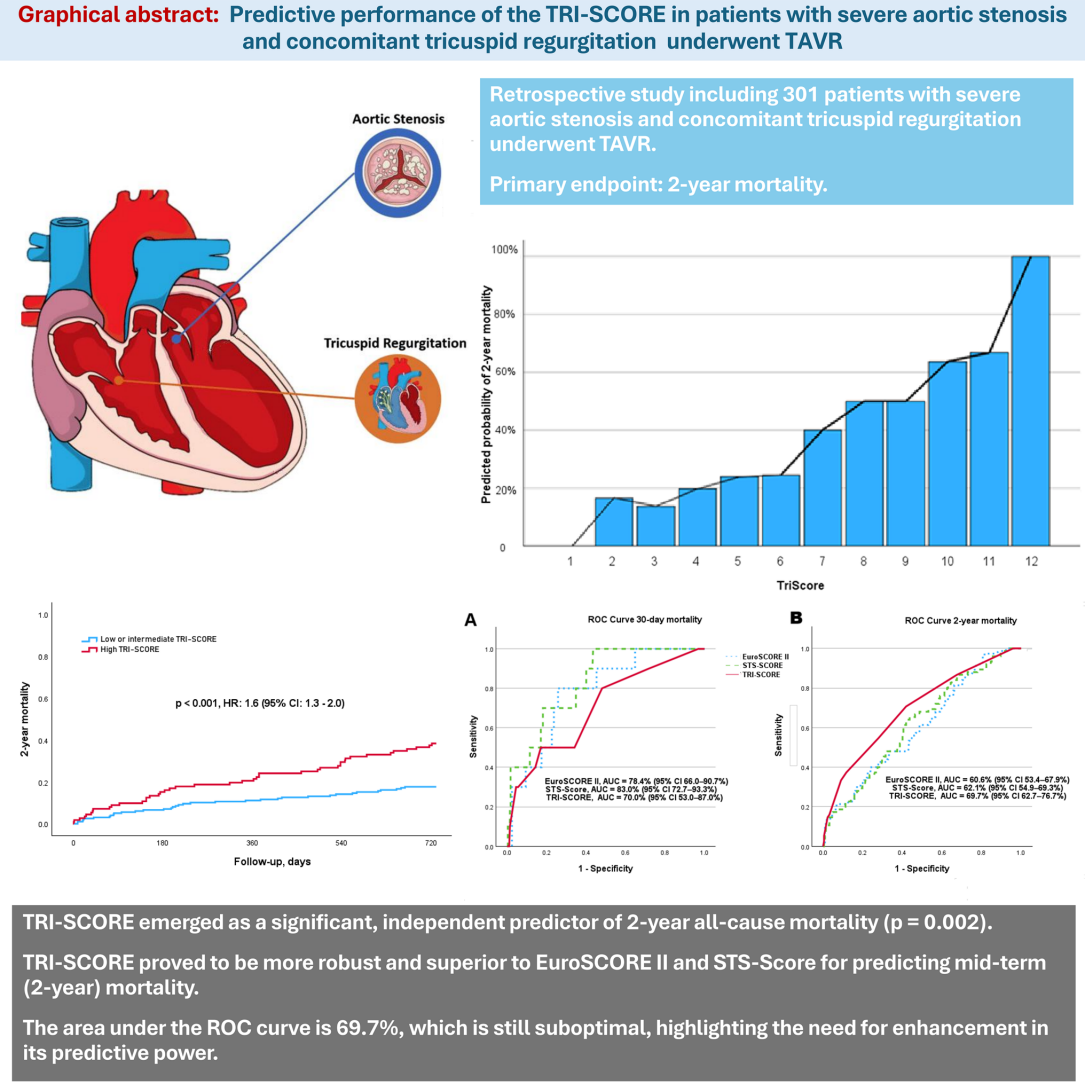

## Introduction

Aortic stenosis (AS) is the most common acquired heart valve disease in the Western world [1]. When symptomatic, the 2-year mortality rate of severe AS is around 50% [2]. Over the last decade, transcatheter aortic valve replacement (TAVR) has emerged as a transformative therapy for most patients with severe symptomatic AS, especially those who are deemed inoperable or are at moderate to high surgical risk [3]. 

Tricuspid regurgitation (TR), often caused by AS and consecutive left-sided heart failure (HF) with secondary postcapillary pulmonary hypertension, is a common valvular condition associated with poor outcomes [4]. Studies have demonstrated a significant increase in mortality for HF patients with TR [5].

Risk stratification is crucial in guiding therapeutic decisions for patients undergoing valve interventions. Several clinical scoring systems, such as the EuroSCORE II and the Society of Thoracic Surgeons score (STS-Score), are routinely used to assess perioperative risk for cardiac surgeries [6, 7].

In September 2021, Dreyfus et al. introduced the TRI-SCORE, a risk model specifically designed to predict in-hospital mortality after tricuspid valve surgery. This model incorporates eight clinical parameters reflecting the severity of the patient's condition, including right ventricular (RV) dysfunction and kidney and liver function [8]. Subsequent studies have validated the TRI-SCORE's utility not only in surgical patients but also in transcatheter and conservatively managed cohorts, with a focus on predicting both in-hospital mortality and mid-term outcomes [9, 10]. Further studies have demonstrated the significant discriminative ability of the TRI-SCORE in predicting mid- and long-term mortality following tricuspid valve repair [11, 12]. The presence of concomitant significant (typically ≥ moderate) tricuspid regurgitation (TR) prior to TAVR is associated with an approximately two-fold increase in both early and mid-term all-cause mortality among patients with aortic stenosis (AS) [13–15]. Although reductions in TR have been observed in patients with severe AS and concomitant TR following TAVR [16], progression of TR may still occur in some patients even after intervention [17]. Therefore, the TRI-SCORE may serve as an additional tool for predicting mid-term mortality in this high-risk cohort, supporting improved risk stratification and informing post-TAVR therapeutic strategies for TR.

For this reason, we aimed to evaluate the applicability of the TRI-SCORE in patients with severe AS and moderate-to-severe TR undergoing TAVR. We sought to analyse the model's predictive value for adverse outcomes in this patient population and to compare its performance with the more commonly used EuroSCORE II and STS-Score.

## Methods

### Patient population

We retrospectively analyzed the data of 301 patients with symptomatic severe AS and moderate or severe TR who underwent TAVR between 2013 and 2022 at the Heart Center Bonn. We included all patients with concomitant TR from our TAVR registry in the analysis. The primary endpoint was the 2-year all-cause mortality, which was assessed during the index hospital stay after procedure. If the endpoint was not already met during follow-up visits, assessment of vital status was done by telephone contact with the patient or general practitioner. No data imputation was performed. Patients who underwent tricuspid valve repair during the follow-up period were excluded from the analysis.

The retrospective use of patient’s data for scientific needs was approved by the local ethics committee and written informed consent was obtained from all patients prior to the procedure, specifically for the procedure itself and the subsequent scientific use of data.

Before the TAVR procedure, all patients underwent a careful evaluation including pre-interventional transthoracic and transesophageal echocardiography (including three-dimensional measurements) and had an interdisciplinary discussion with the local, institutional Heart Team. Details about patient screening, procedural techniques, and adjunctive medication have been described elsewhere [18].

### TRI-SCORE and study endpoints

The TRI-SCORE model is based on eight key parameters, encompassing four clinical factors (age ≥ 70 years, NYHA functional class III–IV, signs of right-sided heart failure, and a daily furosemide dose ≥ 125 mg), two laboratory markers (glomerular filtration rate < 30 mL/min and elevated total bilirubin), and two echocardiographic parameters (left ventricular ejection fraction < 60% and moderate/severe right ventricular dysfunction).

We assessed the TRI-SCORE within our patient population and categorized the cohort into two risk groups based on their total TRI-SCORE: low-to-intermediate risk (score 0–5; *n* = 196) and high risk (score 6–12; *n* = 105). We analyzed and compared baseline characteristics, procedural details, and clinical outcomes between these groups.

Additionally, we assessed the predictive value of the TRI-SCORE for 30-day and 2-year all-cause mortality. Its performance was directly compared to well-established surgical risk scoring systems, including the EuroSCORE II and the STS-Score, to determine their relative effectiveness in predicting outcomes within this specific patient cohort.

### Statistical analysis

Normally distributed data are shown as mean ± standard deviation or as counts with percentages, non-normally distributed data is presented as a median with range or inter-quartile range (IQR). *p* values have been used for comparison using student’s *t*-test, Fisher exact tests or Chi-squared tests as appropriate. Clinical event rates for outcomes are shown with the use of Kaplan–Meier curves and compared with the log-rank test. Individual risk factors for 2-year mortality were evaluated using logistic regression, estimating Hazard ratio’s (HRs) and corresponding 95% confidence intervals. The sensitivity and specificity of the different risk scores were assessed via receiver operating characteristics (ROC) analysis. The degree of separability was determined through AUC (area under the curve) assessment. The diagnostic performance of the TRI-SCORE, the EuroSCORE II and the STS-Score was compared using risk reclassification analysis. DeLong test and Bonferroni correction were used to evaluate predictive performance.

Differences were considered statistically significant when *p* < 0.05. Statistical analyses were conducted with SPSS Statistics version 29.0 (IBM Corporation, Somers, NY, USA). The investigators initiated the study, had full access to the data, and wrote the manuscript. All authors vouch for the data and its analysis.

## Results

A total of 301 patients were included in the present analysis. The patient cohort presented with a mean age of 82.1 ± 6.1 years and an intermediate operative risk (EuroSCORE II: 7.2 ± 6.3, STS-Score: 7.4 ± 7.2). The mean TRI-SCORE for the entire cohort was 4.9 ± 2.0, reflecting an intermediate risk. From the overall cohort, 63 patients (20.9%) had severe TR, whereas 238 patients (79.1%) suffered from moderate TR. Approximately more than the half of the patients were female (55.8%) and all patients underwent transfemoral TAVR. Follow-up outcome data were collected for all 301 patients, with a mean follow-up duration of 621 ± 219 days (median: 730 days; IQR: (703–730) days).

### Baseline and procedural characteristics

Baseline characteristics according to the TRI-SCORE are presented in Table 1. A higher TRI-SCORE was associated with a higher degree of TR (severe TR: low or intermediate risk: 14.3% vs. high risk: 33.3%; *p* < 0.001). Overall, the cohort was highly symptomatic with regard to heart failure symptoms, with 12 patients (8.6%) experiencing dyspnoea at rest (NYHA functional class IV); however, no significant differences were found between the groups (*p* = 0.219). Atrial fibrillation was more prevalent in the high-risk group compared to the low or intermediate-risk group (70.2% vs. 84.8%; *p* = 0.005). Patients with cardiac implantable electronic devices (CIED) were more common in the high-risk group (19.8% vs. 38.1%; *p* = 0.009). In contrast, there were no significant differences between the groups with regard to the presence of chronic obstructive pulmonary disease (COPD; *p* = 0.369), hypertension (*p* = 0.732), diabetes mellitus (*p* = 0.793), coronary artery disease (*p* = 0.379), or prior cardiac surgery (*p* = 0.159).

**Table 1:** Baseline characteristics according to the TRI-SCORE risk groups

	All patients *n* = 301	Low or intermediate risk *n* = 196	High risk *n* = 105	*p*-value
Female sex	168 (55.8)	114 (58.2)	54 (51.4)	0.275
Age	82.1 ± 6.1	81.8 ± 6.3	82.3 ± 6.0	0.497
BMI	26.1 ± 4.9	25.9 ± 4.8	26.0 ± 5.0	0.449
COPD	61 (20.3)	43 (21.9)	18 (17.1)	0.369
Hypertension	258 (85.7)	169 (86.2)	89 (85.7)	0.732
Diabetes mellitus	91 (30.2)	58 (29.6)	33 (31.4)	0.793
NYHA IV	12 (8.6)	5 (6.0)	7 (12.7)	0.219
Previous MI	41 (13.6)	22 (11.2)	19 (18.1)	0.113
Coronary artery disease	192 (63.8)	121 (61.7)	71 (67.6)	0.379
Atrial fibrillation	223 (75.3)	134 (70.2)	89 (84.8)	0.005
Prior cardiac surgery	29 (10.0)	15 (8.1)	14 (13.3)	0.159
CIED	49 (25.9)	25 (19.8)	24 (38.1)	0.009
Dialysis	11 (3.7)	3 (1.6)	8 (7.6)	0.019
eGFR	51.7 ± 17.2	54.7 ± 14.9	44.2 ± 18.8	< 0.001
NT-proBNP, pg/ml	2837 (1553/6912)	2373 (1331/5553)	4961 (2392/12280)	< 0.001
Ejection fraction, %	53.6 ± 12.1	55.8 ± 12.1	49.3 ± 12.0	< 0.001
AVA, cm^2^	0.73 ± 0.18	0.74 ± 0.18	0.71 ± 0.20	0.352
sPAP, mmHg	46.0 ± 15.6	46.0 ± 15.8	47.2 ± 16.2	0.561
Severe TR > II°	63 (20.9)	28 (14.3)	35 (33.3)	< 0.001
EuroSCORE II	7.2 ± 6.3	5.7 ± 4.1	10.1 ± 8.3	< 0.001
STS-Score	7.4 ± 7.2	6.5 ± 6.4	8.3 ± 7.5	0.037
TRI-SCORE	4.9 ± 2.0	3.8 ± 0.9	7.4 ± 1.6	< 0.001
NYHA > III	256 (85.0)	161 (82.1)	95 (90.5)	0.062
Right ventricle dysfunction	104 (34.6)	25 (12.8)	79 (75.2)	< 0.001
Elevated bilirubin	39 (13.0)	6 (3.1)	33 (31.4)	< 0.001
Daily dose of furosemide ≥ 125 mg	59 (19.6)	11 (5.6)	48 (45.7)	< 0.001
Procedural time, min	67.6 ± 34.2	65.4 ± 27.8	73.3 ± 43.0	0.196

Values are mean (± SD), median (IQR 1/3) or *n*/*N* (%)

*BMI* body mass index, *COPD* chronic obstructive pulmonary disease; *NYHA* New York Heart Association; *MI* myocardial infarction; *CIED* cardiac implantable electronic devices; *eGFR* estimated glomerular filtration rate; *NT-proBNP* n-terminal pro brain natriuretic peptide; *AVA* aortic valve area; *sPAP* systolic pulmonary artery pressure; *TR* tricuspid regurgitation

Regarding echocardiographic parameters, no significant differences were observed in aortic valve area (AVA) (*p* = 0.352) or systolic pulmonary artery pressure (sPAP) (*p* = 0.561). However, there was a significant difference with regard to the ejection fraction (EF), with the high-risk group demonstrating a significant lower EF compared to the low or intermediate-risk group (55.8 ± 12.1% vs. 49.3 ± 12.0%; *p* < 0.001). 

Procedural characteristics, such as the amount of contrast media use, fluoroscopy time, and procedure duration, did not differ significantly between the groups. The procedure was successful in all study participants.

### Clinical outcomes

**Fig. 1:**
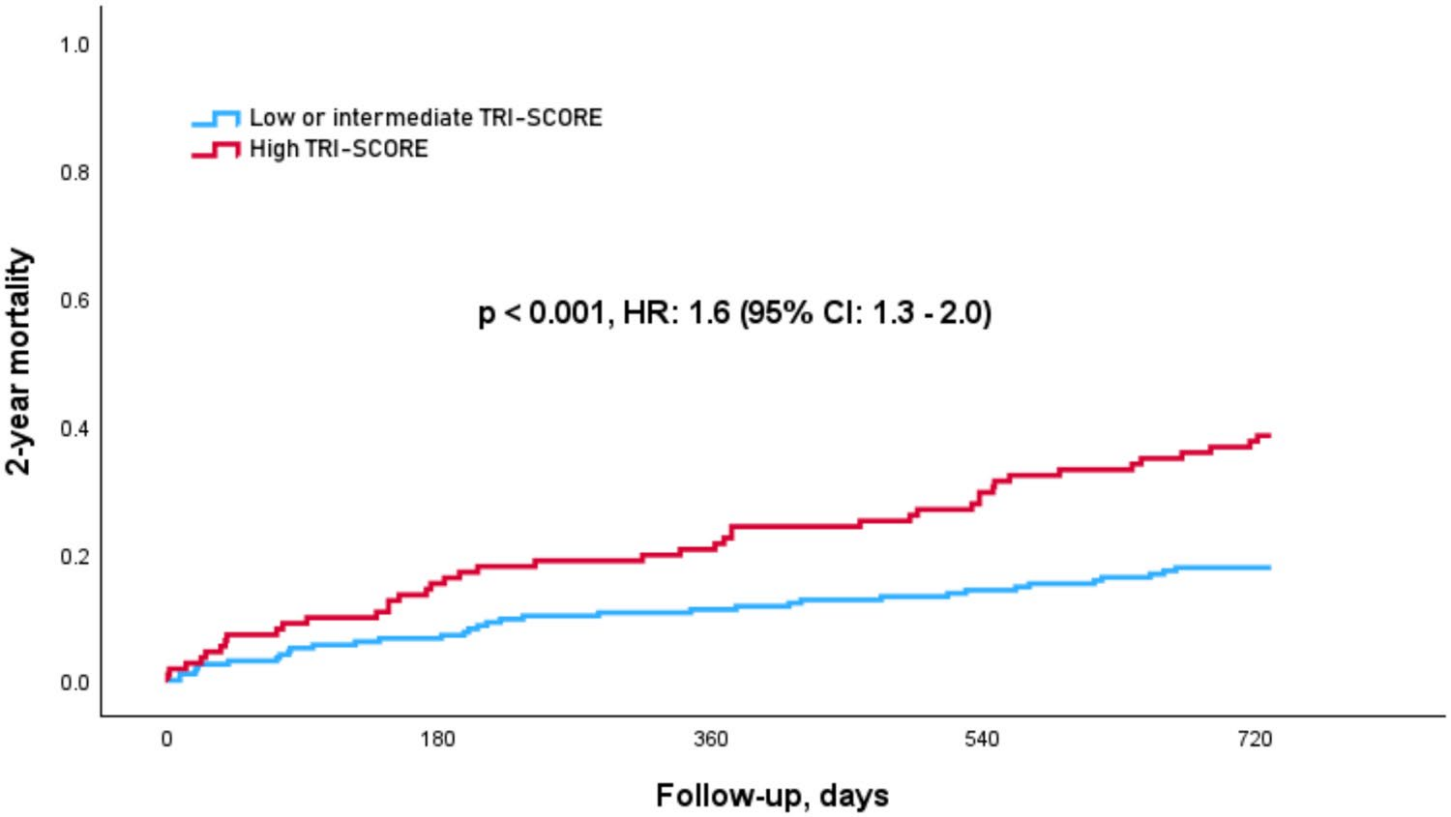
Kaplan–Meier survival analysis of 2-year all-cause mortality, stratified by the TRI-SCORE. Cox regression analysis showed that patients in the high-risk group (> 6) had significantly higher rates of mortality with a hazard ratio (HR) of 1.6 (95% CI 1.3–2.0; *p* < 0.001)

Outcome data according to the TRI-SCORE revealed statistically significant differences in the 2-year all-cause mortality between the groups. Patients in the low or intermediate-risk group had a significantly lower 2-year mortality rate compared to the high-risk group (17.9% vs. 40.0%; *p* < 0.001) with a hazard ratio (HR) of 1.6 (95% CI 1.3–2.0; *p* < 0.001), as shown in Fig. 1. Notably, this difference was primarily driven by the subgroup with moderate TR (*n* = 238), (low or intermediate risk: 16.7% vs. high risk: 42.9%; *p* < 0.001). In contrast, in the subgroup with severe TR (*n* = 63), this difference did not reach statistical significance most likely due to the unequal number of patients across the different TR severity groups (low or intermediate risk: 25.0% vs. high risk: 34.3%; *p* = 0.582). When comparing overall mortality rates across TR severity grades, 2-year mortality increased with TR severity (moderate TR: 24.4% vs. severe TR: 30.2%; *p* = 0.007).

**Fig. 2:**
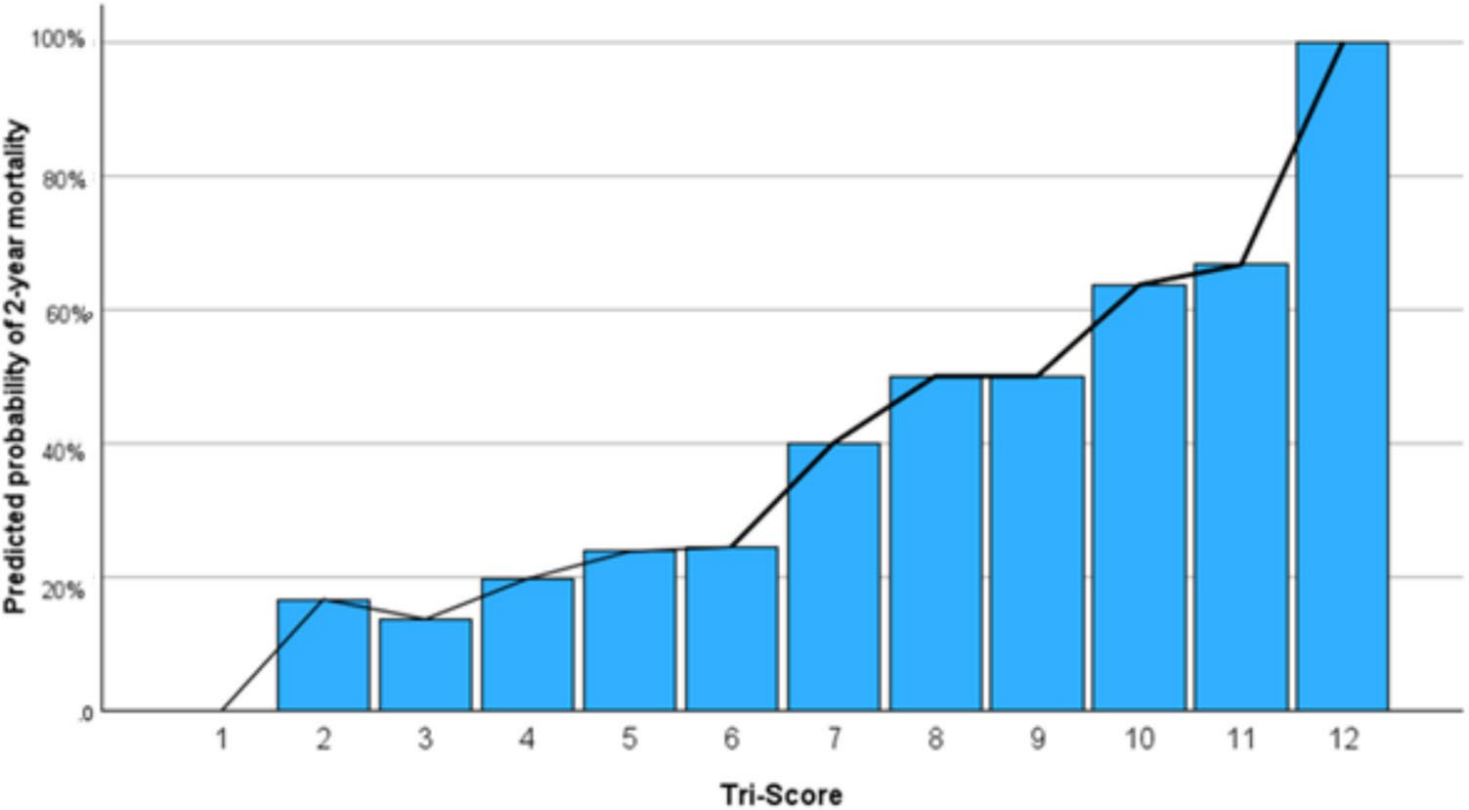
Bar chart, representing the predicted probability of 2-year mortality based on TRI-SCORE values

The predicted probability of 2-year mortality based on TRI-SCORE values is illustrated in Fig. 2, demonstrating the correlation between increased mortality rates and a higher TRI-SCORE. Mortality risk increases progressively with rising TRI-SCORE values, although the correlation is weak (Spearman correlation coefficient: 0.262, *p* < 0.001). In terms of 30-day mortality, the high-risk group exhibited a slightly higher mortality rate; however, this difference was not statistically significant (2.6% vs. 4.8%; *p* = 0.326).

### Multivariate analysis

The multivariate analysis for 2-year mortality demonstrated that the TRI-SCORE was a significant independent predictor of mid-term mortality (HR = 1.4, 95% CI 1.2–1.8; *p* = 0.002). This association remained significant after adjustment for potential confounders. In contrast, several variables that were significant in the univariate analysis lost statistical significance in the multivariate model, as shown in Table 2, including atrial fibrillation (*p* = 0.114), chronic kidney disease (*p* = 0.148). Elevated bilirubin (*p* = 0.599) and signs of right heart failure (*p* = 0.603) were also not significant in the multivariate analysis, most likely due to their inclusion as component parameters of the TRI-SCORE, leading to collinearity within the model. Furthermore, while EuroSCORE II and the STS-Score are established perioperative risk assessment tools, they did not reach statistical significance in predicting 2-year mortality in the multivariate analysis (*p* = 0.674 and *p* = 0.101, respectively).

**Table 2:** Univariate and multivariate analysis with the most important confounders

	Univariate analysis		Multivariate analysis	
	HR (95% CI)	*p* value	HR (95% CI)	*p* value
TRI-SCORE	1.6 (1.3–2.0)	** < 0.001**	1.4 (1.2–1.8)	** < 0.001**
Female sex	0.8 (0.5–1.3)	0.448	–	
Age < 80	0.5 (0.3–0.8)	0.005	0.4 (0.2–0.8)	**0.012**
COPD	1.2 (0.7–1.9)	0.610	–	
Diabetes mellitus	0.8 (0.5–1.4)	0.510	–	
CIED	1.4 (0.8–2.4)	0.244	–	
Coronary artery disease	0.9 (0.6–1.4)	0.664	–	
Atrial fibrillation	2.9 (1.4–6.0)	**0.004**	2.0 (0.8–4.6)	0.114
Chronic kidney disease	2.1 (1.3–3.6)	**0.006**	1.6 (0.8–3.4)	0.148
Elevated Bilirubin	1.5 (1.1–1.9)	**0.004**	0.9 (0.6–1.4)	0.599
LVEF < 50%	1.4 (0.9–2.2)	0.163	–	
Signs of right HF	1.6 (1.3–2.0)	** < 0.001**	1.1 (0.8–1.6)	0.603
TR > II	1.8 (0.8–2.1)	0.329	0.7 (0.3–1.4)	0.245
EuroSCORE II > 8	1.8 (1.2–2.9)	**0.006**	0.9 (0.4–1.8)	0.674
STS-Score > 5	2.1 (1.3–3.3)	**0.002**	1.8 (0.9–3.6)	0.101

*HR* hazard ratio; *CI* confidence interval; *COPD* chronic obstructive pulmonary disease; *CIED* cardiac implantable electronic devices; *LVEF* link ventricular ejection fraction; *TR* tricuspid regurgitation

Bold values indicate statistical significance (*p* < 0.05)

### Performance analysis of the TRI-SCORE

**Fig. 3:**
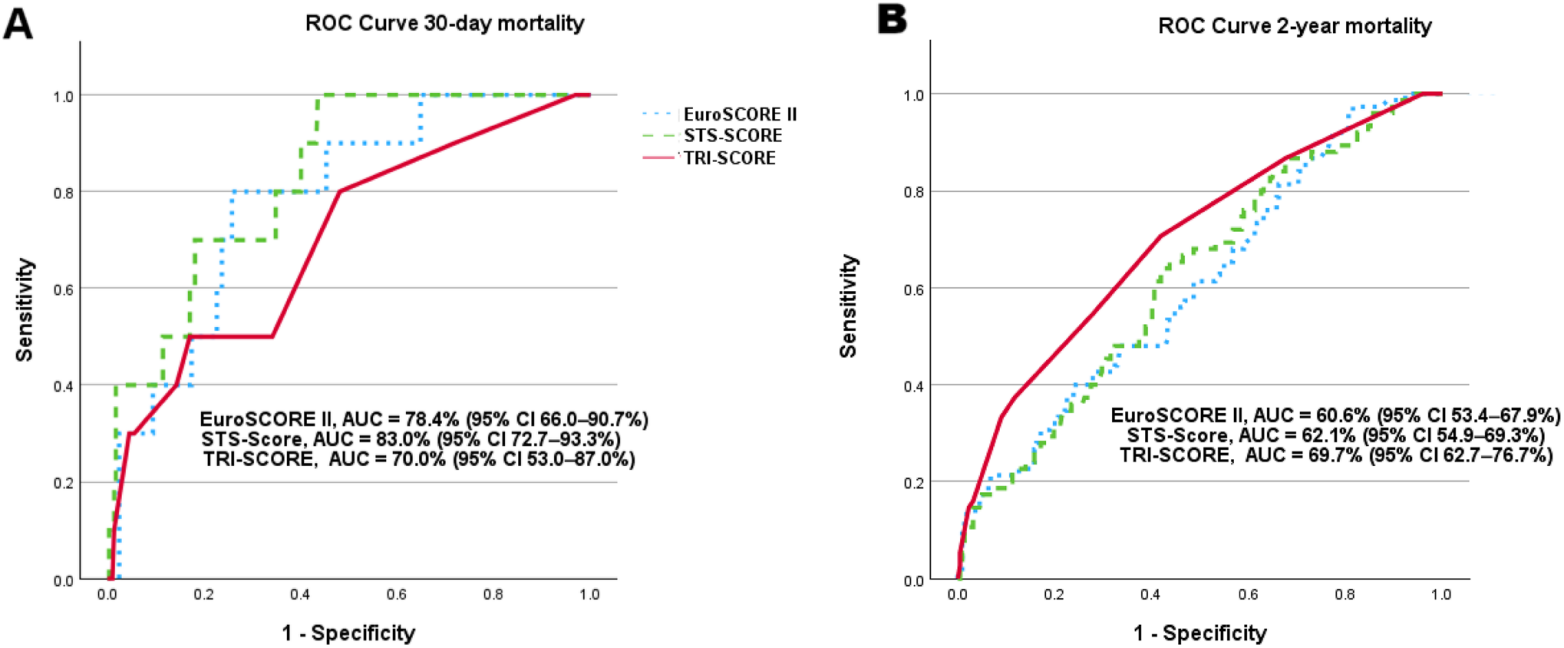
ROC analyses for 30-day—and 2-year all-cause mortality. **A** EuroSCORE II and STS-Score performed better in predicting 30-day mortality compared to the TRI-SCORE. The EuroSCORE II achieved an AUC of 78.4% (95% CI 66.0–90.7%; *p* < 0.001), while the STS-Score showed an AUC of 83.0% (95% CI 72.7–93.3%; *p* = 0.001), In contrast, the TRI-SCORE had a lower AUC of 70.0% (95% CI 53.0–87.0%; *p* = 0.021). **B** TRI-SCORE proved to be more robust and superior to EuroSCORE II and STS-Score in predicting 2-year mortality. The TRI-SCORE achieved an AUC of 69.7% (95% CI 62.7–76.7%; *p* < 0.001), which was higher than the EuroSCORE II (AUC: 60.6%, 95% CI 53.4–67.9%; *p* = 0.004) and the STS-Score (AUC: 62.1%, 95% CI 54.9–69.3%; *p* = 0.001)

ROC analysis of the TRI-SCORE, EuroSCORE II, and STS-Score was conducted for both the 30-day and 2-year follow-up periods. For predicting 30-day mortality, the EuroSCORE II and STS-Score demonstrated a strong predictive value. The EuroSCORE II achieved an AUC of 78.4% (95% CI 66.0–90.7%; *p* < 0.001), while the STS-Score had an AUC of 83.0% (95% CI 72.7–93.3%; *p* = 0.001), as expected both of which were superior to the TRI-SCORE, which had an AUC of 70.0% (95% CI 53.0–87.0%; *p* = 0.021) for 30-day mortality (Fig. 3A). However, the TRI-SCORE emerged numerically as a more robust and reliable tool for predicting 2-year mortality, with an AUC of 69.7% (95% CI 62.7–76.7%; *p* < 0.001), compared to the EuroSCORE II (AUC: 60.6%, 95% CI 53.4–67.9%; *p* = 0.004) and the STS-Score (AUC: 62.1%, 95% CI 54.9–69.3%; *p* = 0.001) (Fig. 3B). However, due to overlapping confidence intervals, these differences were not statistically significant after Bonferroni correction (all Bonferroni-corrected *p* > 0.05; TRI-SCORE vs. EuroSCORE II: *p* = 0.188, TRI-SCORE vs. STS-Score: *p* = 0.102).

Across multiple performance metrics as shown in Table 3, TRISCORE demonstrates the best overall performance, with the highest AUC, Youden index (0.276), and F1-score (43.5), indicating a more balanced and effective predictive tool compared to EuroSCORE II and STS-Score. However, it is important to note that all three models exhibit relatively low positive predictive values (PPV) (TRI-SCORE: 36%, EuroSCORE II: 32%, STS-Score: 28%), otherwise, negative predictive values (NPV) remain relatively high across all models, with TRISCORE reaching 85%, indicating a strong ability to rule out mortality risk when the score is low.

**Table 3:** Performance comparison of TRISCORE, EuroSCORE II, and STS Score in med-term mortality prediction

	TRISCORE	EeroSCORE II	STS-Score
AUC (95% CI)	70.6 (63.8–77.3)	61.3 (54.3–68.4)	60.1(53.1–67.0)
Accuracy	68	68	56
Best cutoff	6	8	5
Youden index	0.276	0.164	0.189
Sensivity	55	41	63
Specificity	72	76	56
F1 score	43.5	35.9	38.8
PPV	36	32	28
NPV	85	81	84

*AUC* area under the curve; *PPV* positive predictive value; *NPV* negative predictive value; *STS Score* Society of Thoracic Surgeons Score

## Discussion

To the best of our knowledge, this is the first study to validate the TRI-SCORE in patients with AS and concomitant moderate-to-severe TR undergoing transcatheter aortic valve replacement (TAVR). The main findings of our study can be summarized as follows:

1. Patients with a high TRI-SCORE (≥ 6) had a significantly higher 2-year mortality rate compared to patients with a low–intermediate TRI-SCORE (< 6).
2. The TRI-SCORE emerged as a significant, independent predictor of 2-year all-cause mortality (*p* = 0.002).
3. The TRI-SCORE proved to be more robust and superior to EuroSCORE II and STS-Score for predicting mid-term (2-year) mortality.
4. The TRI-SCORE showed good discrimination for high-risk patients regarding mid-term outcomes, with an HR of 1.6 (95% CI 1.3–2.0); however, the area under the ROC curve is 69.7%, which is still below 80%, highlighting the need for enhancement in its predictive power.

Advanced TR is a well-recognized comorbidity among patients with severe AS undergoing TAVR [19]. Moreover, moderate to severe TR has been linked to a worse prognosis, including increased long-term mortality rates and a higher likelihood of hospitalization due to heart failure in AS patients [13–15]. Amano et al*.* showed that the 5-year freedom rate from death or hospitalization was significantly lower in patients with coexistent moderate or severe TR compared to those without (49.1% vs. 67.3%; *p* < 0.001), independent of comorbidities and AS severity [20]. In TAVR patients, the presence of more than mild TR prior to the procedure was associated with higher rates of 1-year mortality (25.1% vs. 11.1%; *p* < 0.001) and was an independent predictor of mortality [21]. Although this population represents a highly selected group of the total TAVR cohort, concomitant TR plays a pivotal role in patient outcomes and should be carefully considered during the decision-making process and individual risk stratification. This consideration could also help guide the development of post-TAVR therapeutic strategies for severe TR, such as transcatheter tricuspid valve intervention, which may improve outcomes for these high-risk patients.

The TRI-SCORE was a novel tool to predict in-hospital mortality after isolated tricuspid valve surgery and includes several specific TR-related parameters [8]. In our patient population, including TAVR patients with moderate to severe TR, a TRI-SCORE ≥ 6 was significantly associated with increased 2-year mortality. Even after adjusting for common confounders, the TRI-SCORE remained a significant and independent predictor of mid-term mortality. These findings indicate that the TRI-SCORE is applicable in this selected patient population and has potential utility for risk stratification.

Risk assessment tools, such as the EuroSCORE II and the STS-Score, are widely recognized for their utility in predicting periprocedural and short-term mortality in patients with AS undergoing cardiac procedures [22]. These scores are implemented in the ESC guidelines for valve therapies and provide valuable guidance in clinical decision-making by helping to stratify patients based on procedural risks. The TRI-SCORE, on the other hand, is specifically designed for isolated TR interventions and has been validated in patients undergoing tricuspid valve surgery or catheter-based interventions. It has demonstrated robust performance, particularly in predicting 30-day clinical outcomes, making it a valuable tool for short-term risk stratification in this subset of patients [23]. However, the STS-score and EuroSCORE II are established tools for predicting perioperative risk in TAVR and SAVR patients, so it is not surprising that the TRI-SCORE could not outperform these traditional TAVR risk scores for short-term outcome prediction. Interestingly, the TRI-SCORE excels in its ability to predict mid-term (2 year) outcome in our patient population. This superior accuracy is supported by other studies, which highlight the TRI-SCORE’s enhanced discriminatory power over the EuroSCORE II for forecasting mid-term mortality in patients undergoing TR repair [11, 24]. These findings underline the importance of tailoring risk assessment tools to specific patient populations and conditions, ensuring optimal clinical utility and outcome prediction.

With an AUC of 69.7% (below the 80% threshold), the TRI-SCORE demonstrated moderate but suboptimal performance in predicting clinical events in our study cohort. Research by Adamo et al. has also shown limitations in the TRI-SCORE’s ability to predict clinical outcomes in patients undergoing transcatheter tricuspid valve interventions [25], noting that the TRI-SCORE performed poorly in terms of both discrimination and calibration. In our observation, this may be due to the TRI-SCORE’s primary focus on right-heart parameters, which are only partially relevant for patients with AS, where left-heart pathology is often a major concern. Therefore, a reasonable approach to risk stratification might involve considering both the EuroSCORE II and STS-Scores alongside the TRI-SCORE. This would provide a more comprehensive assessment of patient risk. Additionally, the EuroSCORE III model is currently under development to offer more personalized risk assessments by incorporating advanced clinical variables, such as frailty, to further refine risk predictions.

## Limitations

The primary limitation of this study lies in its retrospective, single-center design, which inherently carries the risk of selection bias and limits the generalizability of the findings to broader populations. Additionally, the relatively small sample size poses another challenge, as it may restrict the statistical power to detect subtle differences or trends. Nonetheless, considering the complexity of managing patients with both AS and concomitant TR undergoing TAVR, the cohort size is adequate for the application and evaluation of the TRI-SCORE risk model. Another important limitation of this study is the considerable heterogeneity in the follow-up period, as indicated by the large standard deviation (219 days) and wide interquartile range (703–730 days). This variability may introduce inconsistencies in outcome assessment and affect the comparability of results. Additionally, differences in follow-up duration among patients could influence the interpretation of long-term survival and event rates. Future studies with larger, multicenter cohorts and prospective designs would be instrumental in addressing these limitations.

## Conclusion

Our study highlights the effectiveness of the TRI-SCORE in predicting mid-term mortality in patients with AS and moderate-to-severe TR undergoing TAVR. While the EuroSCORE II and STS-Score demonstrated superior predictive capability for 30-day mortality, the TRI-SCORE emerged as a more robust tool for mid-term outcomes.

## Abbreviations

AS: Aortic valve stenosis
TAVR: Transcatheter aortic valve replacement
TR: Tricuspid regurgitation
HF: Heart failure
RV: Right ventricular
CIED: Cardiac implantable electronic devices
COPD: Chronic obstructive pulmonary disease
CABG: Coronary artery bypass grafting
AUC: Area Under the curve
TRI-SCORE: Tricuspid regurgitation in-hospital score
EuroSCORE II: European system for cardiac operative risk evaluation II
STS: Society of thoracic surgeons
NYHA: New York heart association
ROC: Receiver operating characteristic
